# Associations between Life’s Crucial 9 and severity, all-cause mortality, and cardiovascular mortality in individuals with cardiovascular-kidney-metabolic syndrome: the mediating role of phenotypic age acceleration

**DOI:** 10.3389/fnut.2025.1612293

**Published:** 2025-08-06

**Authors:** Jing Li, Zengxin Xu, Jiayi Lin, Shunxiang Luo, Shanghua Xu

**Affiliations:** ^1^Department of Clinical Medicine, Fujian Medical University, Fuzhou, Fujian, China; ^2^Department of Cardiology, Nanping First Hospital Affiliated to Fujian Medical University, Nanping, China; ^3^Key Laboratory of Gastrointestinal Cancer, Ministry of Education, School of Basic Medical Sciences, Fujian Medical University, Fuzhou, China

**Keywords:** Life’s Crucial 9, cardiovascular-kidney-metabolic syndrome, phenotypic age acceleration, restricted cubic spline, NHANES

## Abstract

**Objective:**

The American Heart Association (AHA) recently introduced the concept of cardiovascular-kidney-metabolic (CKM) syndrome. This study aimed to explore the associations of Life’s Crucial 9 (LC9), the recently updated lifestyle guidelines for cardiovascular health, with the severity and mortality of CKM syndrome.

**Methods:**

For this study, we utilized National Health and Nutrition Examination Survey (NHANES) data and conducted logistic regression analyses to evaluate the association between LC9 scores and the severity of CKM syndrome, whereas Cox regression models were employed to assess the effects of LC9 scores and mortality in patients with CKM syndrome. Subsequently, restricted cubic spline (RCS) analysis was used to investigate potential nonlinear relationships between LC9 scores and both the severity and mortality of CKM syndrome. Stratification and interaction analyses were carried out across different subgroups to validate the findings. Finally, mediation analysis was performed to investigate whether phenotypic age acceleration (PhenoAgeAccel) status mediates the association between LC9 scores and CKM syndrome severity.

**Results:**

A total of 7,647 patients were included in our study. After adjusting for covariates, each 10% increase in the LC9 score was associated with a 37% reduction in the risk of progression to advanced CKM syndrome stages, and WQS analysis revealed that the blood glucose level was the most influential contributing factor. Moreover, Cox regression analysis revealed that higher LC9 scores were significantly linked to lower risks of all-cause mortality (HR = 0.81, 95% CI: 0.76–0.87) and cardiovascular mortality (HR = 0.74, 95% CI: 0.65–0.84) among individuals with CKM syndrome. Additionally, RCS analysis revealed a significant nonlinear association between LC9 scores and CKM syndrome severity, but no nonlinear association was found between LC9 scores and the risk of mortality. Mediation analysis confirmed that PhenoAgeAccel mediated the association between LC9 scores and CKM syndrome severity.

**Conclusion:**

Our findings indicate significant negative associations of LC9 scores with CKM syndrome severity and mortality, highlighting the potential role of LC9 in guiding targeted public health strategies to prevent the progression of CKM syndrome to advanced stages and reduce mortality risk.

## 1 Introduction

Epidemiological studies have revealed that metabolic diseases, kidney diseases, and cardiovascular diseases (CVDs) frequently coexist, contributing to a substantial burden on global health (1). Recently, the American Heart Association (AHA) (2) introduced the term “cardiovascular–kidney–metabolic (CKM) syndrome” to represent the complex interactions among metabolic syndrome (MetS), CVDs, and chronic kidney disease (CKD). The AHA also emphasized the importance of comprehensive management of these interconnected diseases to prevent potential adverse outcomes.

Health issues associated with CKM syndrome are very common in the general population. An estimated 9–11% of the population is affected by heart disease, while kidney disease and metabolic disorders have prevalence rates of approximately 15 and 13%, respectively, in the United States (3). These diseases share common risk factors, and they amplify each other’s effects, leading to poor prognosis. For example, CVD is closely linked to kidney function, and impaired renal function can lead to elevated blood pressure and volume overload; conversely, CVD can accelerate the progression of kidney dysfunction (4–6). Metabolic disturbances, particularly diabetes and obesity, contribute to IR, endothelial dysfunction, and vascular inflammation, creating a vicious cycle that worsens health outcomes (7, 8). Besides, studies (9–11) have shown that poor CKM health status is related to an increased risk of CVD and worse clinical outcomes, with the combined risk exceeding that of each disease individually. These findings specified the urgency of approaching metabolic, kidney, and cardiovascular components as an integrated system to prevent the progression of CKM syndrome. Notably, the AHA also underlined (2) the significance of identifying individuals in the preclinical stages of CKM and advocated for early research efforts aimed at preventing CVD events and halting disease progression. Therefore, understanding the factors related to CKM syndrome severity and mortality is essential for initiating preventive interventions at an early stage and improving the prognosis of CKM syndrome.

The CKM syndrome staging framework reflects both the progression of CKM syndrome and the increasing absolute risk of CVD. Ndumele et al. (2) suggested that the definition of CKM syndrome should integrate the current concepts of cardiovascular health (such as Life’s Essential 8, LE8) while also recognizing the role of social determinants of health (SDOH) and the importance of promoting individual health. This approach highlights maintaining optimal cardiovascular health to prevent the development of CKM syndrome risk factors. It is noteworthy that some key health metrics within LE8 are fundamental components of CKM syndrome. Numerous observational studies have suggested obvious associations between LE8 and health outcomes such as CKD, CVD, MetS, and diabetes (12–15). Moreover, recent perspectives (16–19) have also increasingly highlighted the critical role of mental health in CKD, CVD, and MetS, and these findings demonstrate that depressive symptoms may be positively linked to CKM syndrome. In 2025, the AHA introduced Life’s Crucial 9 (LC9), a new indicator that integrates depression into the existing LE8 framework. Based on this, we speculate that the LC9 score may be significantly associated with CKM syndrome progression. Unfortunately, no relevant evidence in this field is available thus far. In this study, we aimed to explore the potential associations between LC9 scores and both severity and mortality of CKM syndrome using data from the National Health and Nutrition Examination Survey (NHANES). Additionally, we examined the mediating role of phenotypic age acceleration (PhenoAgeAccel) in the correlation between LC9 scores and CKM syndrome severity to inform more effective risk stratification and timely interventions.

## 2 Materials and methods

### 2.1 Study population

**Figure 1:**
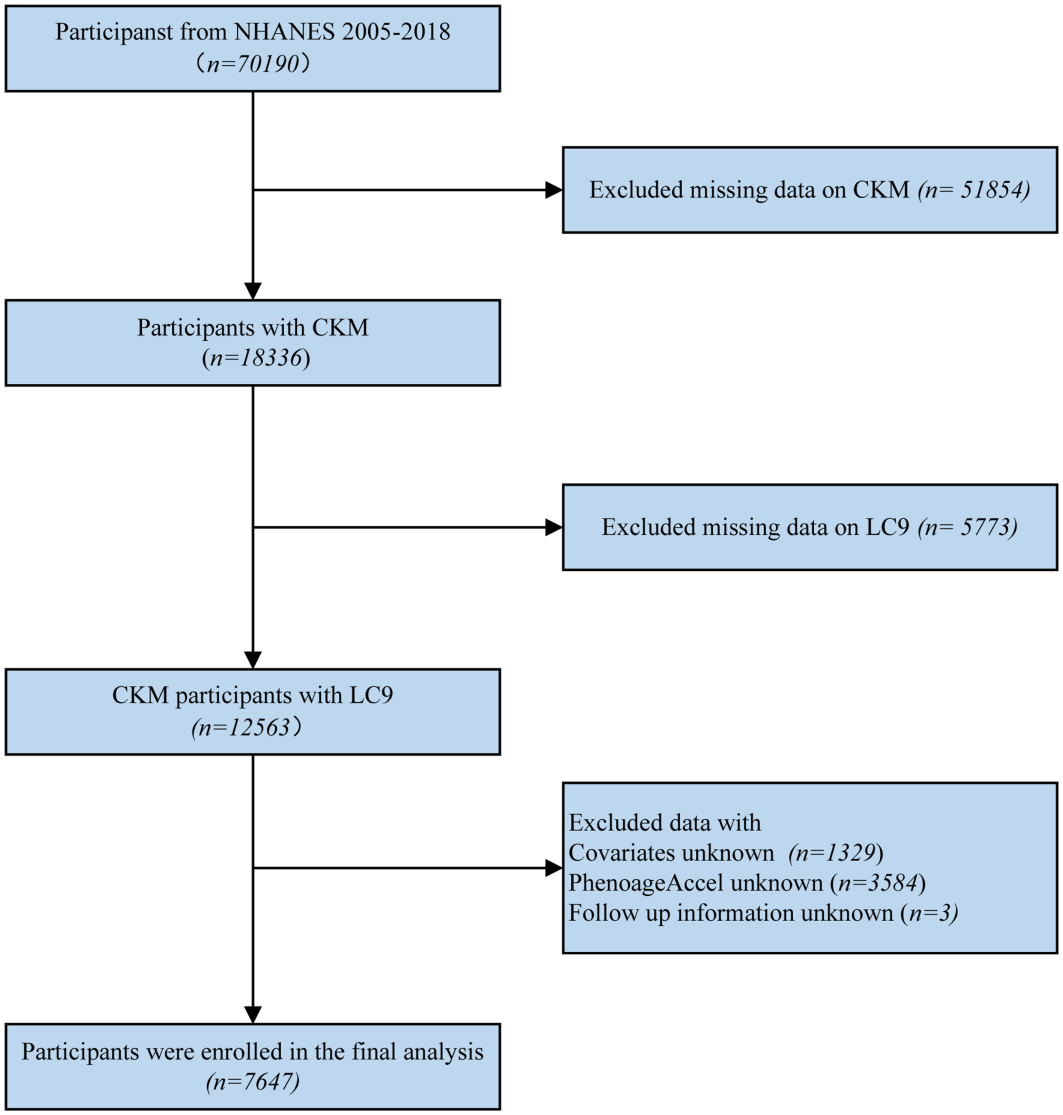
The flowchart of the NHANES database study.

For this research, we utilized the NHANES dataset. Initially, 70,190 participants were included. Individuals with insufficient data to determine CKM syndrome staging (*n* = 51,854), missing LC9 information (*n* = 5,773), missing key covariates (*n* = 1,329), missing data on PhenoAgeAccel (*n* = 3,584), or missing follow-up data (*n* = 3) were excluded. Ultimately, a total of 7,647 participants were included in the final analysis (the details of the inclusion and exclusion process are displayed in Figure 1).

### 2.2 Assessment of CKM syndrome

The classification of CKM syndrome stages was based on data from the NHANES and the criteria outlined in the AHA’s scientific statement. Information was collected through standardized questionnaires and physical examinations, with blood and random urine samples analyzed at a central laboratory. For each participant, CKM syndrome stages were determined following previous studies (Supplementary Table S1) (20, 21). The 10-year cardiovascular risk was calculated using the AHA PREVENT equations, with high risk defined as a 10-year predicted risk of ≥20% (22). In addition, the stages of CKD were assessed using the race-free CKD Epidemiology Collaboration 2021 creatinine equation for estimated glomerular filtration rate (eGFR) and the urinary albumin-to-creatinine ratio (23). The participants were categorized into advanced CKM syndrome (stages 3 or 4) or nonadvanced CKM syndrome (stages 0, 1, or 2) (21).

### 2.3 LC9 assessments

The LC9 score encompasses five health factors (psychological health, blood pressure, blood glucose, non-high-density lipoprotein, and BMI) and four health behaviors (sleep duration, nicotine exposure, physical activity, and diet) (24). Each component is evaluated on a scale ranging from 0 to 100, and the overall LC9 score is derived by averaging the scores of all nine components. A higher LC9 score reflects better cardiovascular health. To assess these components, dietary quality was assessed via the Healthy Eating Index-2015 (HEI-2015), while data on diabetes history, sleep patterns, physical activity levels, smoking status, medication use, and depression scores (assessed using the Patient Health Questionnaire-9 [PHQ-9]) were collected through self-report questionnaires (25). Additionally, the NHANES dataset included physical examinations to measure participants’ blood pressure, height, and weight, as well as blood sample analyses to assess glycated hemoglobin, blood glucose levels, and lipid profiles. Life’s Simple 7 (LS7) encompasses seven domains: nicotine exposure, BMI, physical activity, total cholesterol, blood pressure, blood glucose, and diet. In comparison, LE8 extends this framework by incorporating sleep health and updates the algorithms for several existing metrics. Building upon this, LC9 further adds psychological health as an additional component (26).

### 2.4 Assessments of PhenoAgeAccel

PhenoAge, which reflects biological aging, was estimated through the analysis of blood chemistry parameters in clinical laboratory tests following the PhenoAge algorithm. The calculation of PhenoAge involves 9 biomarkers: C-reactive protein, albumin, glucose, and creatinine levels; white blood cell count; mean cell volume; red cell distribution width; red cell distribution width; and chronological age (27). The selection of these biomarkers was guided by the set utilized in the initial study. PhenoAgeAccel was calculated as the residuals from regressing PhenoAge on chronological age (28).

### 2.5 Mortality data and definition

The National Center for Health Statistics (NCHS) identifies cardiovascular mortality based on classification codes I00-I09, I11, I13, and I20-I51 from the International Classification of Diseases (ICD-10).

### 2.6 Assessment of covariates

Covariates were collected using standardized questionnaires and included age, gender, race, education level, marital status, poverty-income ratio (PIR), and alcohol consumption. Education level was categorized into three groups: individuals with less than a high school education, those who graduated from high school, and those who pursued education beyond the high school level (29). Alcohol consumption was categorized as heavy, moderate, mild, former, or never drinkers, while PIR was classified into three ranges: less than 1.3, between 1.3 and 3.5, and greater than 3.5 (23). Marital status was divided into three groups: individuals who were married or living with a partner; those who had never been married; and individuals who were widowed, divorced, or separated, encompassing those who had previously been married or were cohabiting but were no longer with their partner due to death, legal separation, or personal circumstances (30).

### 2.7 Statistical analysis

Statistical analyses were performed with R software (4.3.1). For continuous variables, weighted means along with standard errors were utilized, whereas categorical variables were summarized using weighted frequency percentages. First, logistic regression analysis was conducted to assess the association between the LC9 level and CKM syndrome severity (from non-advanced to advanced stages). Moreover, for individuals with CKM, Cox regression models were applied to evaluate the effect of LC9 on mortality among individuals with CKM. We established three models to minimize potential confounding effects. In addition, weighted quantile sum (WQS) regression models were employed to examine the link between the individual LC9 components and CKM syndrome severity, and the WQS index was calculated to identify the primary contributing factors. Restricted cubic spline (RCS) models were also constructed to investigate the dose–response associations between LC9 scores and both severity and mortality of CKM. Subgroup and interaction analyses were performed to examine the relationship between LC9 and CKM across various demographic characteristics, including age, gender, race, and education. The E value quantifies unmeasured confounding variables to assess the reliability of the results. Finally, the R package “Mediation” was implemented to investigate the mediating effect of accelerated biological aging on the association between LC9 scores and the severity of CKM syndrome. Receiver Operating Characteristic (ROC) curves, Net Reclassification Improvement (NRI), and Integrated Discrimination Improvement (IDI) were used to compare the incremental predictive value of the LC9, LE8, and LS7 models. *p* < 0.05 was considered to indicate statistical significance.

## 3 Results

### 3.1 Baseline characteristics

As presented in Table 1, a total of 7,647 participants were included in the analysis, including 6,352 individuals with non-advanced CKM syndrome and 1,295 individuals with advanced CKM syndrome. Significant differences were observed between the advanced and non-advanced groups in terms of age, gender, race, educational level, marital status, PIR, alcohol consumption, total LC9 score, the nine components of LC9, and PhenoAgeAccel.

**Table 1:** Characteristics of participants in NHANES among two different groups of CKM syndrome.

Variable	Total	Non-advanced CKM	Advanced CKM	*p-*value
	7,647	6,352 (87.99)	1,295 (12.01)	
Age				<0.001
<60	5,033 (74.23)	4,789 (80.93)	244 (25.13)	
≥60	2,614 (25.77)	1,563 (19.07)	1,051 (74.87)	
Gender				<0.001
Female	3,887 (51.27)	3,356 (52.27)	531 (44.00)	
Male	3,760 (48.73)	2,996 (47.73)	764 (56.00)	
Race				<0.001
Non-Hispanic Black	1,444 (9.40)	1,200 (9.31)	244 (10.08)	
Non-Hispanic White	3,699 (72.55)	2,909 (71.72)	790 (78.65)	
Other Hispanic	685 (4.47)	594 (4.65)	91 (3.13)	
Other race	577 (6.16)	529 (6.43)	48 (4.16)	
Mexican American	1,242 (7.42)	1,120 (7.89)	122 (3.97)	
Education				<0.001
Less than high school	1,734 (13.73)	1,325 (12.59)	409 (22.05)	
High school	1,806 (24.04)	1,475 (23.50)	331 (28.05)	
More than high school	4,107 (62.23)	3,552 (63.91)	555 (49.90)	
Marital status				<0.001
Never married	1,241 (16.03)	1,183 (17.77)	58 (3.30)	
Widowed/divorced/separated	1,680 (17.92)	1,212 (15.99)	468 (32.03)	
Married/living with partner	4,726 (66.05)	3,957 (66.24)	769 (64.67)	
PIR				<0.001
<1.3	2,094 (17.38)	1,674 (16.57)	420 (23.30)	
1.3–3.5	3,073 (37.01)	2,482 (35.98)	591 (44.51)	
>3.5	2,480 (45.61)	2,196 (47.44)	284 (32.19)	
Alcohol consumption				<0.001
Never	958 (9.97)	781 (9.68)	177 (12.06)	
Former	1,245 (13.14)	872 (11.49)	373 (25.22)	
Mild	2,751 (39.42)	2,241 (38.67)	510 (44.84)	
Moderate	1,203 (17.78)	1,092 (19.03)	111 (8.60)	
Heavy	1,490 (19.69)	1,366 (21.12)	124 (9.28)	<0.001
LC9				
Continuous	71.24 (0.29)	72.34 (0.29)	63.16 (0.49)	<0.001
Quantile				<0.001
Q1	1,935 (21.21)	1,361 (18.38)	574 (41.89)	
Q2	1,997 (24.37)	1,623 (23.85)	374 (28.16)	
Q3	1,848 (25.99)	1,624 (26.91)	224 (19.28)	
Q4	1,867 (28.43)	1,744 (30.86)	123 (10.66)	
PhenoAgeAccel (years)	−3.54 (0.09)	−3.96 (0.09)	−0.47 (0.24)	<0.001
HEI diet score	37.78 (0.61)	37.46 (0.64)	40.12 (1.28)	0.046
Physical activity score	71.80 (0.77)	74.04 (0.83)	55.39 (1.94)	<0.001
Tobacco exposure score	70.64 (0.78)	71.04 (0.81)	67.69 (1.61)	0.043
Sleep health score	84.17 (0.45)	84.80 (0.46)	79.55 (1.02)	<0.001
Body mass index score	60.79 (0.59)	61.56 (0.66)	55.13 (1.19)	<0.001
Blood lipid score	65.35 (0.50)	65.60 (0.57)	63.45 (0.82)	0.043
Blood glucose score	87.52 (0.33)	89.86 (0.30)	70.39 (1.15)	<0.001
Blood pressure score	70.80 (0.56)	74.03 (0.58)	47.19 (1.25)	<0.001
Psychological health score	92.30 (0.24)	92.68 (0.25)	89.50 (0.80)	<0.001

### 3.2 Association between LC9 scores and severity of CKM syndrome

**Figure 2:**
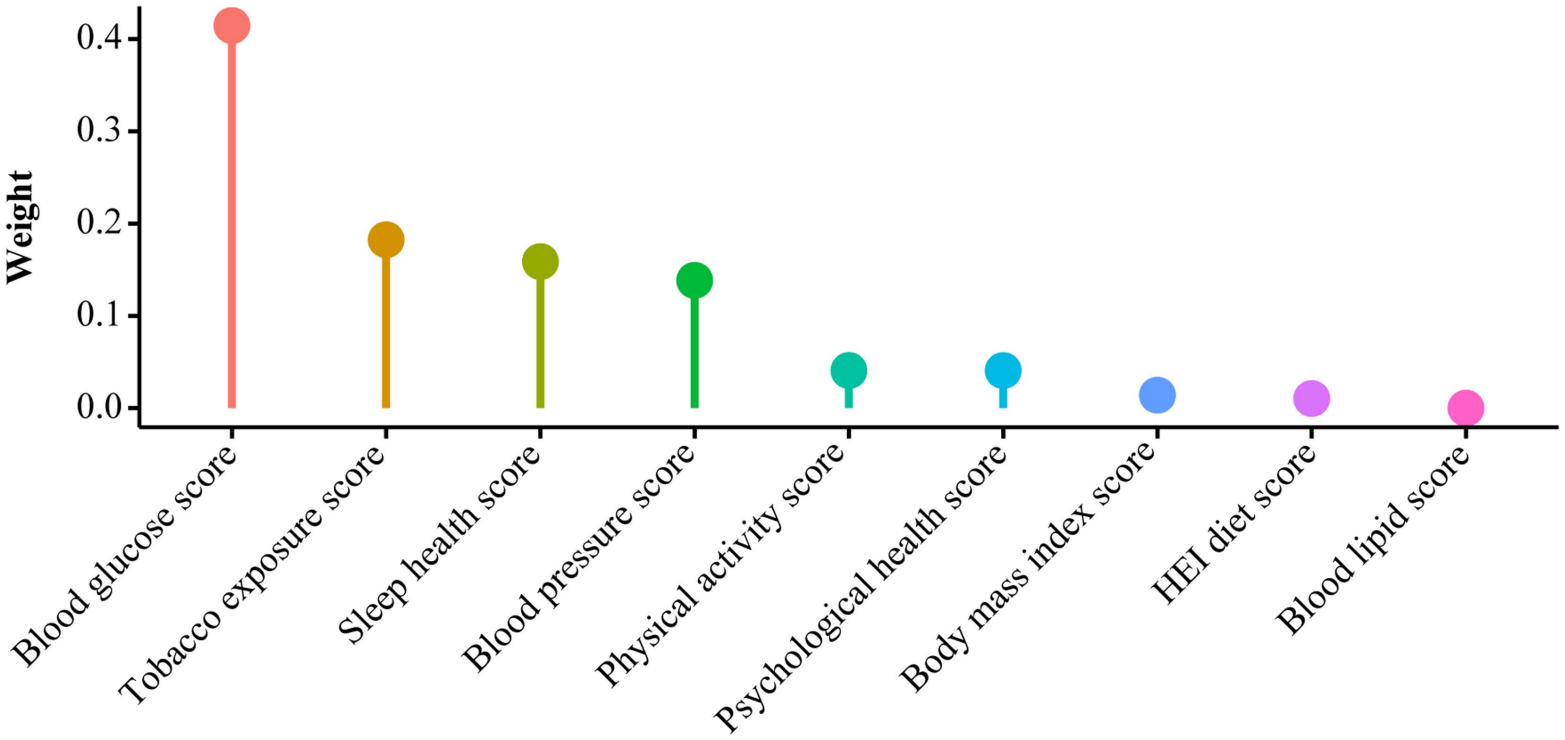
WQS model regression index weights for CKM syndrome, adjusted for age, gender, race, education, marital status, PIR, and alcohol consumption.

As presented in Table 2, all three models consistently revealed an inverse association between the LC9 score and CKM syndrome severity (Model 1: OR = 0.58, 95% CI: 0.55–0.62; Model 2: OR = 0.59, 95% CI: 0.54–0.63; Model 3: OR = 0.63, 95% CI: 0.59–0.68). In Model 3, after adjusting for various covariates, each 10% increase in the LC9 score was associated with a 37% reduction in the risk of progression to advanced CKM syndrome stages. When LC9 was categorized into quartiles, participants in the highest quartile (Q4) had a 75% lower risk of progression to advanced CKM syndrome than did those in the lowest quartile (Q1) [OR = 0.25, 95% CI: 0.19–0.32]. Additionally, the WQS model revealed a negative correlation between the WQS index and the risk of advanced (stages 3–4) CKM syndrome (OR = 0.18, 95% CI: 0.13–0.26, *p* < 0.001). As illustrated in Figure 2 and Supplementary Table S2, the blood glucose level contributed the most to the overall effect.

**Table 2:** Associations between LC9 and the severity of CKM syndrome.

Variable	Model 1		Model 2		Model 3	
	OR (95% CI)	*p*	OR (95% CI)	*p*	OR (95% CI)	*p*
LC9						
Continuous (per 10 scores)	0.58 (0.55,0.62)	<0.001	0.59 (0.54, 0.63)	<0.001	0.63 (0.59, 0.68)	<0.001
Quantile						
Q1	*Ref*		*Ref*		*Ref*	
Q2	0.52 (0.42,0.64)	<0.001	0.47 (0.37, 0.59)	<0.001	0.52 (0.41, 0.65)	<0.001
Q3	0.31 (0.25,0.40)	<0.001	0.30 (0.23, 0.40)	<0.001	0.36 (0.28, 0.47)	<0.001
Q4	0.15 (0.11,0.20)	<0.001	0.20 (0.15, 0.26)	<0.001	0.25 (0.19, 0.32)	<0.001
*P* for trend		<0.001		<0.001		<0.001

Model 1, Unadjusted model; Model 2, Adjusted for age, gender, race; Model 3, Adjusted for age, gender, race, education, marital status, PIR, and alcohol consumption.

### 3.3 Association between LC9 scores and the prognosis of CKM syndrome

**Figure 3:**
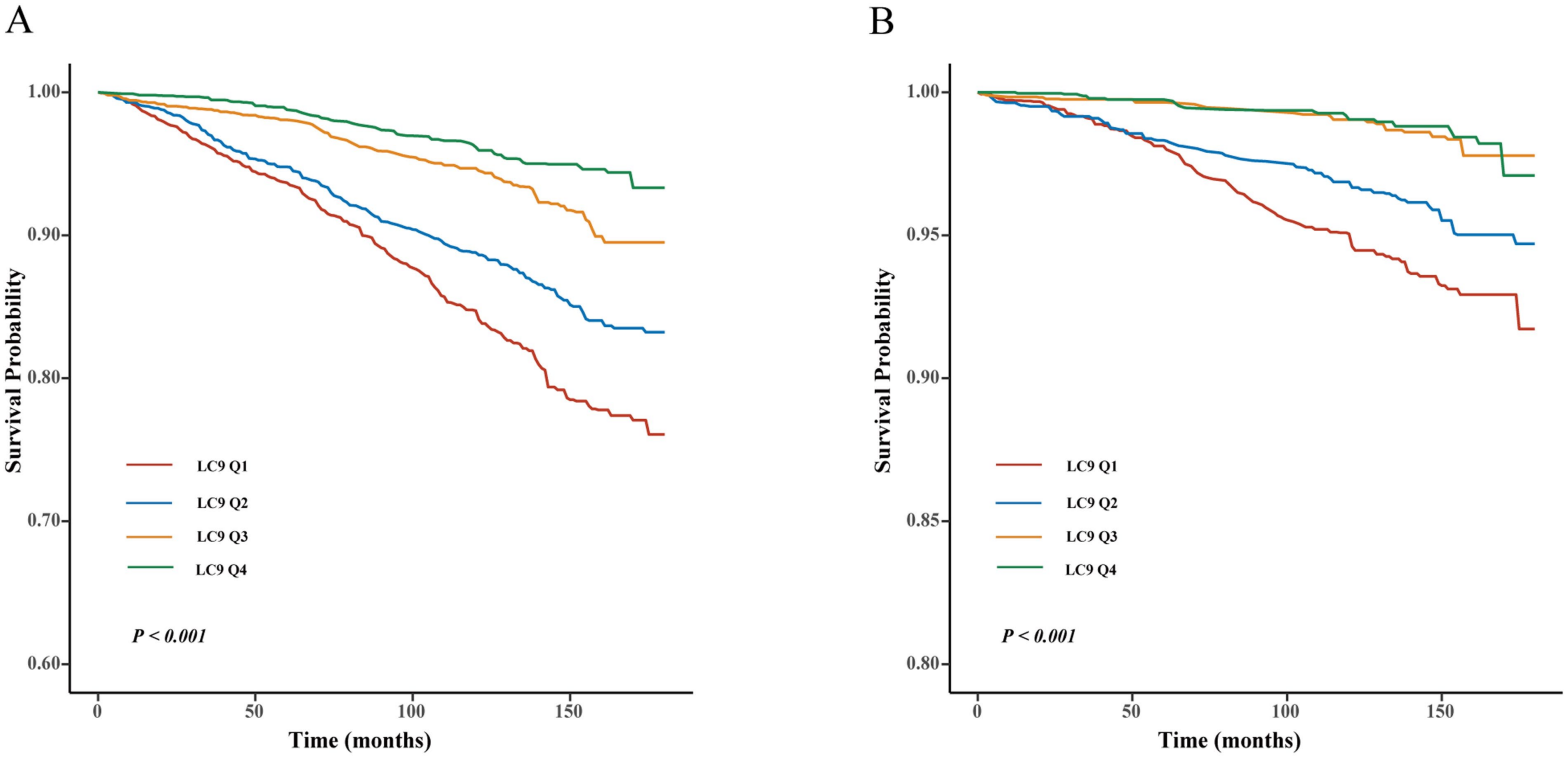
Kaplan–Meier survival curve of LC9 on survival prognosis among individuals with CKM syndrome. **(A)** All-cause mortality; **(B)** cardiovascular mortality.

Cox regression models and Kaplan–Meier (KM) survival curve analysis were employed to evaluate the effect of LC9 on survival prognosis among individuals with CKM syndrome. As illustrated in Figure 3, KM analysis revealed that all-cause and cardiovascular mortality were significantly lower in those with higher LC9 scores in the population with CKM syndrome (*p* < 0.001). As shown in Table 3, all three models consistently suggested that the LC9 score was associated with a reduced risk of all-cause mortality (Model 1: HR = 0.67, 95% CI: 0.63–0.71; Model 2: HR = 0.72, 95% CI: 0.67–0.77; Model 3: HR = 0.81, 95% CI: 0.76–0.87) and cardiovascular mortality (Model 1: HR = 0.62, 95% CI: 0.56–0.68; Model 2: HR = 0.66, 95% CI: 0.58–0.75; Model 3: HR = 0.74, 95% CI: 0.65–0.84).

**Table 3:** Associations between LC9 and the mortality of CKM syndrome.

Variable	Model 1		Model 2		Model 3	
	HR (95%CI)	*p*	HR (95%CI)	*p*	HR (95%CI)	*p*
All-cause mortality						
LC9						
Continuous (per 10 scores)	0.67 (0.63,0.71)	<0.001	0.72 (0.67, 0.77)	<0.001	0.81 (0.76, 0.87)	<0.001
Quantile						
Q1	*Ref*		*Ref*		*Ref*	
Q2	0.70 (0.59,0.84)	<0.001	0.76 (0.65, 0.88)	<0.001	0.87 (0.74, 1.01)	0.073
Q3	0.37 (0.29,0.47)	<0.001	0.44 (0.35, 0.57)	<0.001	0.57 (0.45, 0.72)	<0.001
Q4	0.22 (0.18,0.28)	<0.001	0.35 (0.27, 0.44)	<0.001	0.51 (0.39, 0.67)	<0.001
*P* for trend		<0.001		<0.001		<0.001
Cardiovascular mortality						
LC9						
Continuous (per 10 scores)	0.62 (0.56,0.68)	<0.001	0.66 (0.58, 0.75)	<0.001	0.74 (0.65, 0.84)	<0.001
Quantile						
Q1	*Ref*		*Ref*		*Ref*	
Q2	0.68 (0.47,0.98)	0.040	0.72 (0.50, 1.05)	0.092	0.81 (0.57, 1.17)	0.267
Q3	0.24 (0.15,0.39)	<0.001	0.28 (0.17, 0.47)	<0.001	0.36 (0.23, 0.56)	<0.001
Q4	0.21 (0.14,0.31)	<0.001	0.33 (0.21, 0.52)	<0.001	0.47 (0.28, 0.77)	0.003
*P* for trend		<0.001		<0.001		<0.001

Model 1, Unadjusted model; Model 2, Adjusted for age, gender, race; Model 3, Adjusted for age, gender, race, education, marital status, PIR, and alcohol consumption.

### 3.4 RCS

**Figure 4:**
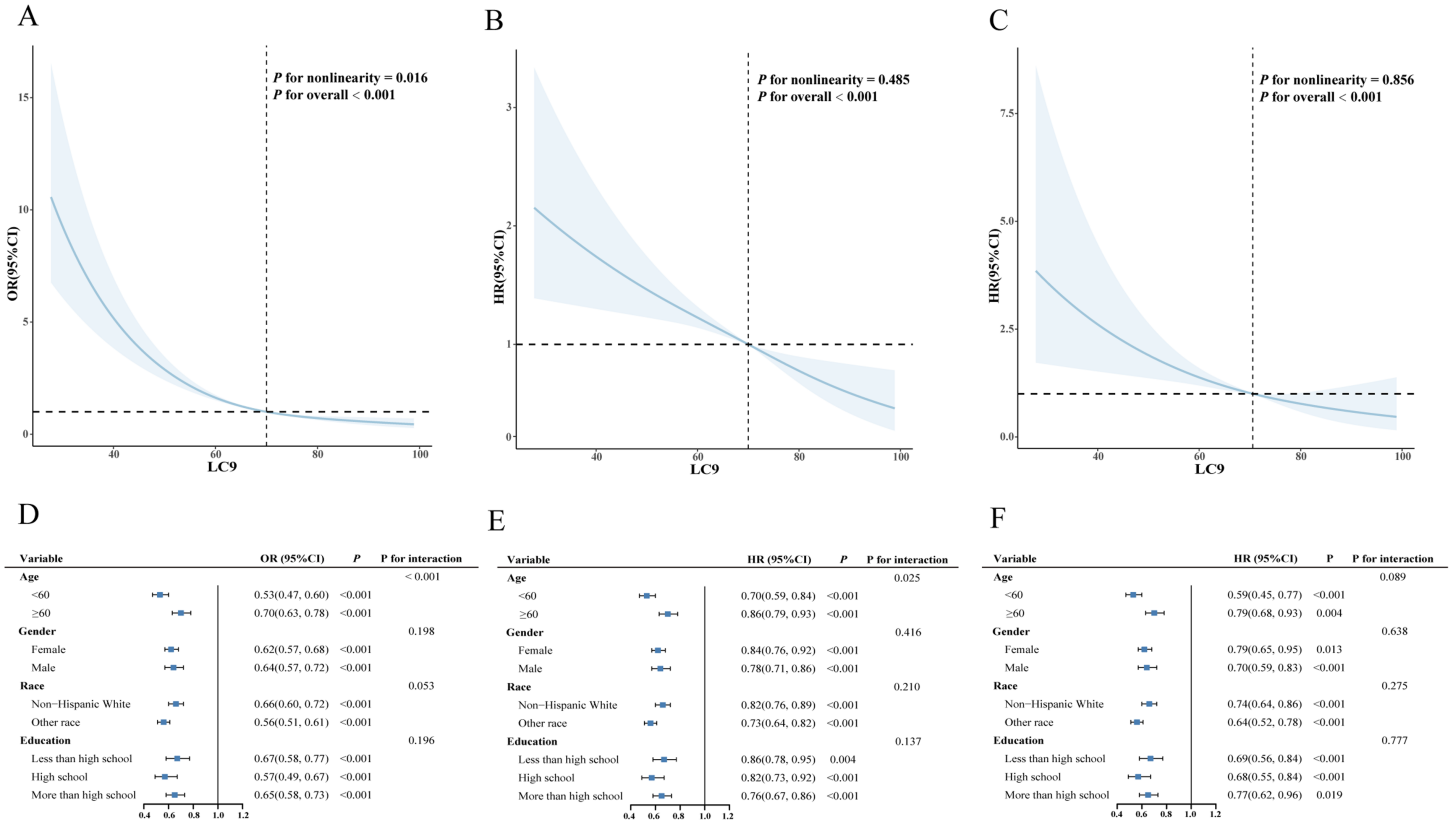
Associations between LC9 and both severity and mortality of CKM syndrome. **(A)** Restricted cubic spline curves for the associations between LC9 and the severity of CKM syndrome. **(B)** Restricted cubic spline curves for the associations between LC9 and all-cause mortality of CKM syndrome. **(C)** Restricted cubic spline curves for the associations between LC9 and cardiovascular mortality of CKM syndrome. **(D)** Subgroup analysis for the association between LC9 and the severity of CKM syndrome. **(E)** Subgroup analysis for the association between LC9 and all-cause mortality of CKM syndrome. **(F)** Subgroup analysis for the association between LC9 and cardiovascular mortality of CKM syndrome.

Additionally, we further explored the effects of LC9 scores on disease progression and prognosis among individuals with CKM syndrome using RCS analysis. As shown in Figure 4A, after adjusting for covariates, a significant nonlinear association was observed between LC9 scores and CKM syndrome severity (*P* for overall < 0.001, *P* for nonlinearity = 0.016). Figures 4B,C demonstrate no nonlinear associations between LC9 scores and either all-cause mortality (*P* for overall < 0.001, *P* for nonlinearity = 0.485) or cardiovascular mortality (*P* for overall < 0.001, *P* for nonlinearity = 0.856) in the CKM syndrome population.

### 3.5 Stratified analysis

As shown in Figures 4D–F, analyses stratified by age, sex, education level, and race revealed significant negative associations between LC9 scores and both the severity and all-cause mortality risk of CKM syndrome. Furthermore, a significant interaction was observed between LC9 scores and age (*p* < 0.05).

### 3.6 Sensitivity analysis

As shown in Supplementary Tables S3, S4, we conducted a sensitivity analysis using the E value to assess whether unmeasured confounders influenced the relationships between LC9 scores and CKM syndrome severity and mortality. The E value indicates that our results remain robust.

### 3.7 Mediation analysis

**Figure 5:**
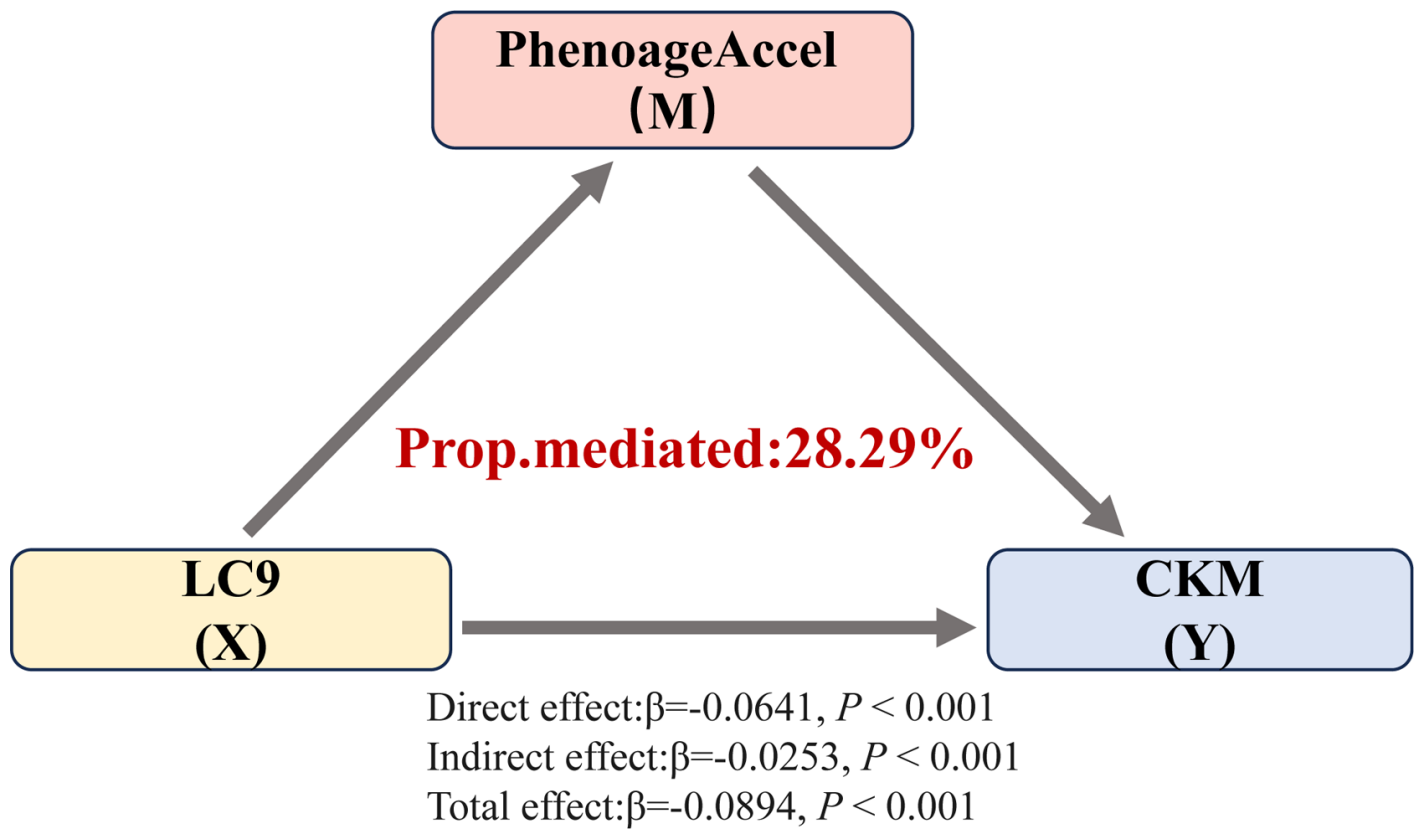
Mediating effect of PhenoAgeAccel on the association between LC9 and the severity of CKM syndrome. Prop.mediated indicates the proportion of mediated effects (proportion of the total effect due to the mediator).

These findings indicated a strong negative association between LC9 scores and the severity of CKM syndrome, with variations observed across different age groups. Therefore, we further explored the mediating effect of PhenoAgeAccel on the relationship between LC9 scores and the severity of CKM syndrome through three potential pathways. As shown in Figure 5, PhenoAgeAccel mediated 28.29% of the relationship between LC9 scores and the severity of CKM syndrome.

### 3.8 Comparison of the associations of LC9, LE8, and LS7 with CKM syndrome

The associations of LE8 and LS7 with CKM syndrome are presented in Table S5. In all analyses, both LE8 and LS7 scores were strongly and inversely associated with CKM syndrome. Notably, after adjustment for multiple covariates, the ORs for the severity of CKM syndrome associated with LS7, LE8, and LC9 scores were 0.77 (95% CI: 0.74–0.80), 0.66 (95% CI: 0.62–0.71), and 0.63 (95% CI: 0.59–0.68), respectively. The HRs for all-cause mortality among individuals with CKM syndrome were 0.88 (95% CI: 0.85–0.92) for LS7, 0.83 (95% CI: 0.78–0.88) for LE8, and 0.81 (95% CI: 0.76–0.87) for LC9. Similarly, the HRs for cardiovascular mortality were 0.82 (95% CI: 0.75–0.89) for LS7, 0.76 (95% CI: 0.68–0.84) for LE8, and 0.74 (95% CI: 0.65–0.84) for LC9. Compared to LS7 and LE8, LC9 showed a significantly stronger inverse association with both CKM severity and mortality.

Given that the scoring algorithms for several variables differ between LS7 and both LE8 and LC9, we constructed a covariate-only baseline model for comparison (31, 32)^.^ As shown in Table 4, the incremental predictive values of adding LS7, LE8, and LC9 to the baseline model were evaluated for both the severity and mortality of CKM syndrome. When predicting the risk of progression to advanced CKM syndrome stages, the NRI increased to 0.464, 0.427, and 0.465, and the IDI increased to 0.026, 0.027, and 0.030, for LS7, LE8, and LC9, respectively. In prognostic models, where the median follow-up time was used as the cutoff for calculating IDI and NRI, LC9 showed no substantial improvement in either metric compared to LE8 and LS7. Furthermore, since LC9 builds upon LE8 by adding only one additional component, psychological health, we further assessed the incremental predictive value of LC9 using LE8 as the baseline model (24, 33). As shown in Table 5, LC9 significantly improved the prediction of CKM progression compared to LE8 (*p* < 0.001), whereas no significant improvement was observed for CKM-related mortality. These findings suggest that, compared to LS7 and LE8, LC9 may provide greater incremental predictive value for the progression to advanced CKM syndrome stages. However, its predictive performance for CKM-related mortality remains comparable across the three models.

**Table 4:** Incremental predictive values of LS7, LE8, and LC9 for both the severity and mortality of CKM syndrome.

Model	NRI (95% CI)	*p*	IDI (95% CI)	*p*
CKM severity				
Basic model	*Ref*		*Ref*	
Basic model + LS7	0.464 (0.405–0.522)	<0.001	0.026 (0.020–0.031)	<0.001
Basic model + LE8	0.427 (0.368–0.485)	<0.001	0.027 (0.021–0.033)	<0.001
Basic model + LC9	0.465 (0.406–0.523)	<0.001	0.030 (0.024–0.036)	<0.001
All-cause mortality				
Basic model	*Ref*		*Ref*	
Basic model + LS7	0.141 (0.093–0.191)	<0.001	0.009 (0.003–0.017)	<0.001
Basic model + LE8	0.140 (0.093–0.186)	<0.001	0.009 (0.003–0.016)	<0.001
Basic model + LC9	0.156 (0.101–0.198)	<0.001	0.009 (0.003–0.016)	<0.001
Cardiovascular mortality				
Basic model	*Ref*		*Ref*	
Basic model + LS7	0.242 (0.153–0.327)	<0.001	0.019 (0.007–0.040)	0.002
Basic model + LE8	0.185 (0.115–0.292)	<0.001	0.014 (0.004–0.031)	0.002
Basic model + LC9	0.213 (0.126–0.304)	<0.001	0.014 (0.004–0.031)	0.004

The basic model included the following covariates: age, gender, race, education, marital status, PIR, and alcohol consumption.

**Table 5:** Incremental predictive values of adding psychological health score to LE8 for both the severity and mortality of CKM syndrome.

Model	NRI	*p*	IDI	*p*
CKM severity				
LE8	*Ref*		*Ref*	
LE8 + PHQ-9 score	0.151 (0.096–0.206)	<0.001	0.004 (0.002–0.007)	0.001
All-cause mortality				
LE8	*Ref*		*Ref*	
LE8 + PHQ-9 score	0.034 (−0.154–0.077)	0.336	0.000 (−0.001–0.002)	0.699
Cardiovascular mortality				
LE8	*Ref*		*Ref*	
LE8 + PHQ-9 score	0.029 (−0.045–0.107)	0.539	0.000 (0–0.005)	0.440

In further analysis, ROC curve comparisons were conducted (Supplementary Figures S1, S2). Consistent with the previous findings, LC9 demonstrated improved discriminative ability for predicting CKM progression compared to the LE8 model (*p* < 0.05). Although the difference in discriminative performance between LC9 and LS7 for CKM severity did not reach statistical significance, an overall upward trend was observed. Additionally, LC9 showed slightly higher area under the curve (AUC) values for CKM-related mortality than both LE8 and LS7; however, these differences were not statistically significant. Overall, LC9 outperformed LE8 and LS7 in terms of AUC, NRI, and IDI for predicting CKM severity, suggesting improvements in global discrimination, individual risk stratification, and average predictive accuracy. These results indicate that LC9 may offer superior predictive performance for CKM syndrome severity, whereas no clear advantage was observed in its ability to improve the prediction of CKM-related mortality when compared with LE8 and LS7.

## 4 Discussion

This pioneering cross-sectional study revealed a strong negative association between LC9 scores and both the severity and mortality of CKM syndrome in the general population, highlighting the significance of early surveillance and management of CVH.

The negative association was consistent with previous findings. April-Sanders (34) reported that patient education and support for adopting healthier lifestyles are crucial strategies for improving CKM health outcomes. A study based on a mouse model of CKM syndrome observed that the dietary supplement Flexovital (containing extracts of *Rhodiola rosea* and beetroot) significantly reduced elevated blood pressure and damage caused by endothelial-dependent vasodilation, demonstrating notable protective effects (35). Another cross-sectional analysis involving 45,460 participants in the United States reported an association between a higher healthy eating index and lower incidence and mortality rates of CKM syndrome (36). Similarly, blood glucose levels, BMI, and blood pressure are significantly associated with the occurrence of IR, systemic inflammation, and oxidative stress, all of which result in the progression of CKM syndrome (12, 13, 37, 38). These factors are expected to serve as focal points for risk assessment and the development of targeted interventions.

In addition, the results from the WQS regression indicated that blood glucose levels were considered the primary determinant affecting the progression of CKM syndrome. As a common contributing factor to CVD, MetS, and renal dysfunction, blood glucose may indirectly contribute to the progression of CKM syndrome. For example, poor blood glucose regulation has been linked to a more rapid deterioration of kidney function in individuals with diabetic nephropathy (20, 39). A 10-year follow-up trial from a prospective diabetes study in the United Kingdom also emphasized that intensive glucose control could reduce the risk of myocardial infarction and all-cause mortality (40). Moreover, sleep health, nicotine exposure, blood pressure, physical activity, and other components of LC9 also contribute to the progression of CKM syndrome. A large body of research has demonstrated that compliance with current physical activity guidelines, maintenance of good sleep hygiene, blood pressure control, and avoidance of nicotine exposure can lower the likelihood of developing chronic conditions such as CVD and diabetes while promoting overall health (41). Interestingly, depression is also a critical factor in the progression of CKM syndrome. Previous studies have revealed independent associations between depression and metabolic disorders, CVD, and CKD (16, 17, 19). For example, a case–control study conducted among U.S. veterans with diabetes (42) showed that diabetic patients with depression faced an increased risk of developing CKD and CVD compared to those without depression. Our findings reveal the urgent need to integrate mental health care into public health strategies to slow the progression of chronic diseases such as CKM syndrome.

In our study, subgroup analyses suggested a significant difference in the association between LC9 scores and the severity of CKM across different age groups. PhenoAgeAccel, an index that more accurately reflects an individual’s biological aging process than chronological age, has been identified as a risk factor for the incidence and mortality of various diseases, including diabetes, renal dysfunction, and CVD (43–46). Hence, we further explored the mediating role of PhenoAgeAccel in the correlation between LC9 scores and the severity of CKM syndrome. The improvement of lifestyle has been shown to significantly improve biological aging. For instance, studies by Liu et al. and Yang et al. (36, 41) have demonstrated that good health behaviors (including higher LE8 scores and better mental health) may help prevent and slow biological aging. Given these results, we propose that higher LC9 scores may prevent the progression of CKM syndrome by alleviating PhenoAgeAccel.

Furthermore, the predictive utility of LC9 was compared with that of LE8 and LS7 in evaluating CKM syndrome. The findings indicated that all three scores demonstrated good predictive performance for both the severity and mortality of CKM syndrome, with LC9 showing a stronger inverse association with CKM syndrome. Notably, LC9 provided superior predictive performance for identifying individuals at higher risk of CKM severity; however, whether this improvement translates into tangible clinical benefits remains to be further investigated. Meanwhile, LC9 did not show a statistically significant improvement in model performance compared with LE8 and LS7 in predicting the prognosis of CKM syndrome. These results suggest that while LC9 may be preferable for assessing CKM severity, its added value for predicting mortality outcomes is limited. Accordingly, the selection of LC9, LE8, or LS7 in clinical or research settings may depend on the specific outcome of interest, contextual factors, and the availability of relevant data. Considering that LE8 integrates sleep health and LC9 further expands this framework by including psychological health evaluation, these additions may play a meaningful role in the context of CKM syndrome, particularly among specific subpopulations such as individuals with poor sleep quality or underlying mental health issues. This is supported by the results of the weighted quantile sum (WQS) regression, which showed that sleep and psychological health contributed 15.9 and 4.1%, respectively, to CKM progression. In contrast, LS7 was originally designed as a more streamlined tool focusing on general health improvement goals. However, it must be acknowledged that reliance on self-reported data for sleep and psychological health introduces potential biases and measurement limitations. These include susceptibility to subjective interpretation, incomplete coverage of relevant symptoms (including sleep quality and depressive features), and the lack of standardized quantification of symptom severity (47, 48)^.^ Such limitations may undermine the predictive utility of LC9 and highlight the need for more comprehensive assessment approaches to capture these domains accurately. Therefore, future research should explore the use of alternative tools with higher accuracy and greater objectivity to better integrate depression and other health factors into the CVH framework, to manage CKM syndrome. In conclusion, by integrating sleep and psychological health dimensions, LC9 provides a more comprehensive view of health factors relevant to CKM syndrome. In the future, it is also necessary to investigate the cost-effectiveness and practical applicability of LC9, LE8, and LS7 in different clinical settings, as well as their value in different populations and countries, to support wider implementation.

The strengths of this study are obvious. First, as a relatively novel biomarker, LC9 integrates mental health into cardiovascular health assessment for the first time, providing a more comprehensive prognostic evaluation. By employing CKM syndrome as the outcome variable, this study systematically evaluates the intricate interplay between MetS, CKD, and CVD, offering potential avenues for improving CKM syndrome prevention strategies. Meanwhile, although additional independent cohort studies are needed to validate the results and establish broader generalizability, the results are derived from a large cross-sectional NHANES survey dataset representing the U.S. population, which enhances their reliability. However, certain limitations should be recognized. The cross-sectional design restricts the ability to draw causal conclusions, and residual confounding may persist owing to the inability to account for all potential covariates influencing the outcomes. Furthermore, certain CKM health indicators are based on self-reported data, which could result in misclassification and recall bias. Despite these limitations, this study highlights the role of LC9 in the progression and prognosis of CKM syndrome, providing valuable insights for more comprehensive investigations in the future.

## 5 Conclusion

In summary, our study demonstrated significant protective associations between LC9 scores and both the severity and mortality of CKM syndrome, highlighting the potential clinical relevance of LC9 in preventing disease progression. Integrating LC9 into health management strategies may be beneficial for preventing the progression of CKM syndrome to advanced stages and reducing mortality risk.

## Data Availability

Publicly available datasets were analyzed in this study. This data can be found at: www.cdc.gov/nchs/nhanes/.
