# Supplementary material for: Associations between Life’s Crucial 9 and severity, all-cause mortality, and cardiovascular mortality in individuals with cardiovascular-kidney-metabolic syndrome: the mediating role of phenotypic age acceleration
Source: Front Nutr. 2025 Aug 6;12:1612293. doi: 10.3389/fnut.2025.1612293 (PMC12364689; doi:10.3389/fnut.2025.1612293)

Supplementary Material

Supplementary Table S1. Detailed algorithm for evaluating each CKM stage.

CKM stages	Definition
Stage 0: No CKM risk factors	normal body mass index (BMI) (<23 kg/m2 for individuals with Asian ethnicity and <25 kg/m2 for other racial and ethnic groups), normal waist circumference (<80 and <90 cm for women and men with Asian race, respectively, and <88 and <102 cm for women and men in all other race and ethnicity categories, respectively) who did not meet criteria for the other stages.
Stage 1: Excess or dysfunctional adiposity	overweight/obesity (BMI⩾23 kg/m2 for individuals with Asian race and >25 kg/m2 for all other race and ethnic groups), abdominal obesity (waist circumference⩾80 and ⩾90 cm for women and men with Asian race, respectively, and ⩾88 and ⩾102 cm for women and men in other race and ethnicity categories, respectively), or prediabetes (defined as a glycated hemoglobin of 5.7% to <6.5% or a fasting blood glucose of 100 mg/dL to <126 mg/dL).
Stage 2: Metabolic risk factors and CKD	additional metabolic risk factors (triglycerides ⩾135 mg/dL, hypertension, metabolic syndrome, and diabetes or moderate- to high-risk chronic kidney disease.
Stage 3: Subclinical CVD in CKM	very high-risk chronic kidney disease in KDIGO classification or elevated 10-year predicted CVD risk.
Stage 4: Clinical CVD in CKM	established CVD (coronary heart disease, angina, heart attack, heart failure, and stroke). Atrial fibrillation and peripheral artery disease were not included, as these data were not available.

Note: Metabolic syndrome was defined based on the presence of at least three of the following criteria: elevated waist circumference (⩾88 cm for women and ⩾102 cm for men), low high-density lipoprotein cholesterol (HDL) levels (<40 mg/dL for men and <50 mg/dL for women), fasting serum triglycerides ⩾150 mg/dL, elevated blood pressure (systolic ⩾130 mmHg, diastolic ⩾80 mmHg, or the use of antihypertensive medications), or prediabetes.

Supplementary Table S2. WQS model regression index weights for CKM syndrome, adjusted for age, gender, race, education, marital status, PIR, and alcohol consumption.

Variable	Weight
**Blood glucose score**	0.415
**Tobacco exposure score**	0.182
**Sleep health score**	0.159
**Blood pressure score**	0.138
**Physical activity score**	0.041
**Psychological health score**	0.041
**Body mass index score**	0.014
**HEI diet score**	0.010
**Blood lipid score**	0.000

Supplementary Table S3. E-values for the effect of LC9 on the severity of CKM syndrome (and its upper limit of 95% CI) in a fully adjusted model.

E-Value for OR Estimate	E-Value for Upper limit of 95%CI	Variable	Level	Group of CKM	OR (95% CI)
2.55	2.30	LC9	Continuous (per 10 scores)	advanced vs non-advanced	0.63 (0.59, 0.68)
2.12	1.79	LC9	Q2 vs Q1	advanced vs non-advanced	0.52 (0.41, 0.65)
5.00	3.68	LC9	Q3 vs Q1	advanced vs non-advanced	0.36 (0.28, 0.47)
7.46	5.70	LC9	Q4 vs Q1	advanced vs non-advanced	0.25 (0.19, 0.32)

Supplementary Table S4. E-values for the effect of LC9 on the mortality of CKM syndrome (and its upper limit of 95% CI) in a fully adjusted model.

E-Value for HR Estimate	E-Value for Upper limit of 95%CI	Variable	Level	Group	HR (95% CI)
**All-cause mortality**					
1.77	1.56	LC9	Continuous (per 10 scores)	deceased vs alive	0.81(0.76, 0.87)
2.90	2.12	LC9	Q3 vs Q1	deceased vs alive	0.57(0.45, 0.72)
3.33	2.35	LC9	Q4 vs Q1	deceased vs alive	0.51(0.39, 0.67)
**Cardiovascular mortality**					
2.04	1.67	LC9	Continuous (per 10 scores)	deceased vs alive	0.74(0.65, 0.84)
5.00	2.97	LC9	Q3 vs Q1	deceased vs alive	0.36(0.23, 0.56)
3.68	1.92	LC9	Q4 vs Q1	deceased vs alive	0.47(0.28, 0.77)

Supplementary Table S5: Associations of LS7, LE8 and LC9 with the severity and mortality of CKM syndrome.

Variable	Model 1		Model 2		Model 3	
	**OR or HR (95%CI)**	***P***	**OR or HR (95%CI)**	***P***	**OR or HR (95%CI)**	***P***
**Advanced CKM**						
LS7	0.69(0.67,0.71)	<0.001	0.75(0.72, 0.78)	<0.001	0.77(0.74, 0.80)	<0.001
LE8	0.60(0.57,0.64)	<0.001	0.62(0.58, 0.67)	<0.001	0.66(0.62, 0.71)	<0.001
LC9	0.58(0.55,0.62)	<0.001	0.59(0.54, 0.63)	<0.001	0.63(0.59, 0.68)	<0.001
**All-cause mortality**						
LS7	0.75(0.72,0.77)	<0.001	0.83(0.80, 0.87)	<0.001	0.88(0.85,0.92)	<0.001
LE8	0.68(0.65,0.72)	<0.001	0.75(0.71, 0.79)	<0.001	0.83(0.78, 0.88)	<0.001
LC9	0.67(0.63,0.71)	<0.001	0.72(0.67, 0.77)	<0.001	0.81(0.76, 0.87)	<0.001
**Cardiovascular mortality**						
LS7	0.69(0.65,0.74)	<0.001	0.78(0.72, 0.84)	<0.001	0.82(0.75, 0.89)	<0.001
LE8	0.63(0.57,0.69)	<0.001	0.68(0.62, 0.76)	<0.001	0.76(0.68, 0.84)	<0.001
LC9	0.62(0.56,0.68)	<0.001	0.66(0.58, 0.75)	<0.001	0.74(0.65, 0.84)	<0.001

Note: Model 1: Unadjusted model; Model 2: Adjusted for age, gender, race; Model 3: Adjusted for age, gender, race, education, marital status, PIR, and alcohol consumption.

Supplementary Figure S1: Receiver operating characteristic (ROC) curves of cardiovascular health scores for the prediction of the severity of CKM syndrome.

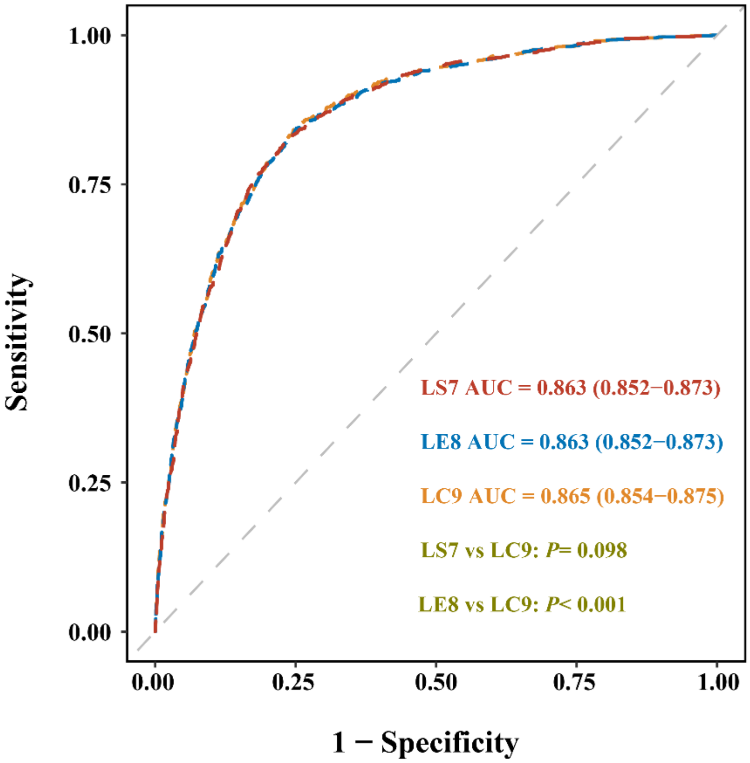

Supplementary Figure S2: Receiver operating characteristic (ROC) curves of cardiovascular health scores for the prediction of mortality. ROC Curves for All-cause mortality (Panels A-C) and Cardiovascular mortality (Panels D-F).

ROC for All-cause Mortality

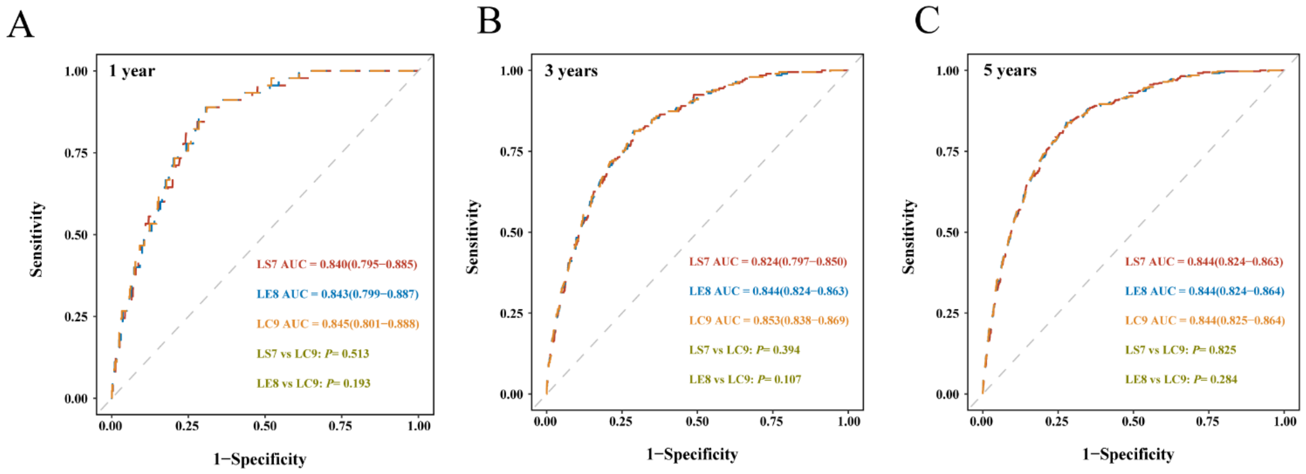

ROC for CVD Mortality

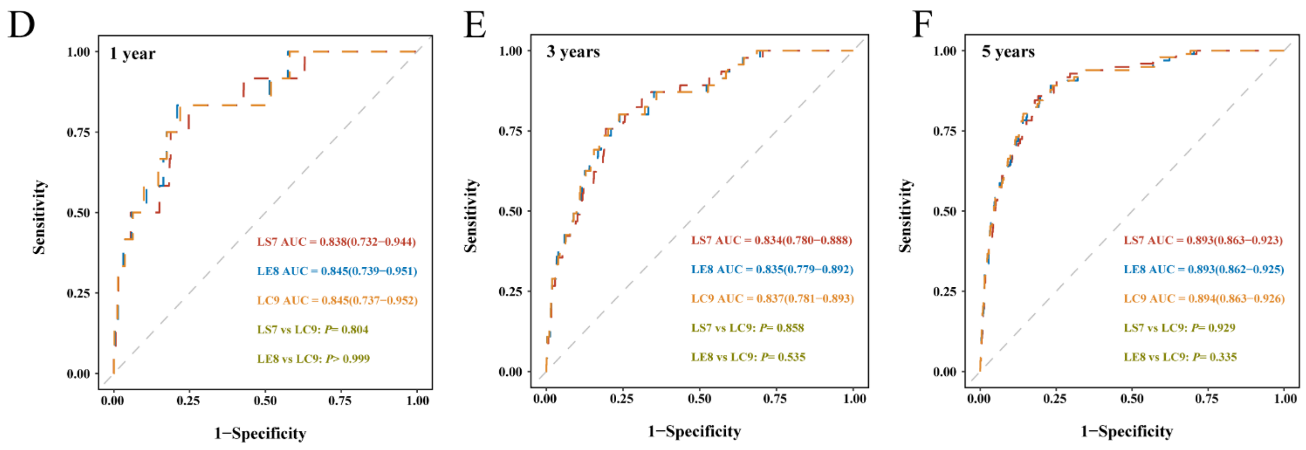

Supplement: Supplementary file 1 [file Data_Sheet_1.pdf]
